# Achievement and Challenges in Orthohantavirus Vaccines

**DOI:** 10.3390/vaccines13020198

**Published:** 2025-02-17

**Authors:** Shiqi Chai, Limei Wang, Hong Du, Hong Jiang

**Affiliations:** 1 Center for Diagnosis and Treatment of Infectious Diseases, TangDu Hospital, The Fourth Military Medical University, Xi’an 710038, China; 18820134730@163.com; 2 Department of Microbiology and Pathogenic Biology, School of Basic Medicine, The Fourth Military Medical University, Xi’an 710032, China; wanglim@fmmu.edu.cn

**Keywords:** vaccine, orthohantavirus, hemorrhagic fever with renal syndrome, hantavirus pulmonary syndrome

## Abstract

Orthohantaviruses (also known as hantaviruses) are pathogens that cause two distinct, yet related forms of severe human disease: hemorrhagic fever with renal syndrome (HFRS) and hantavirus pulmonary syndrome (HPS). These diseases pose a significant threat to global public health due to their high case fatality rates, which can range from 1% to 50%. In recent years, an increasing number of countries and regions have reported human cases, underscoring the urgent need for improved understanding, prevention, and treatment strategies. Given the severity of these diseases and the lack of specific post-exposure antiviral treatments, preventive measures are critical. For several decades, substantial efforts have been dedicated to developing orthohantavirus vaccines, leading to significant advancements. The first large-scale deployment involved inactivated vaccines, which played a crucial role in reducing HFRS incidence in South Korea and China. Subunit vaccines, viral vector vaccines, and virus-like particle (VLP) vaccines have also been extensively researched. Nucleic acid vaccines, including both mRNA and DNA vaccines, hold the greatest potential for future development due to their rapid design and production cycles, ability to elicit robust immune responses, ease of storage and transportation, and adaptable production platforms. Ongoing advancements in computer technology and artificial intelligence promise to further enhance the development of more effective orthohantavirus vaccines.

## 1. Introduction

The *Orthohantavirus* genus belongs to the *Hantaviridae* family within the *Elliovirales* order, and has been recognized as comprising over 35 distinct species [1]. These species have a documented presence across the world, except for Antarctica [2]. Orthohantaviruses primarily cause two fatal human diseases, hemorrhagic fever with renal syndrome (HFRS) and hantavirus pulmonary syndrome (HPS) [3]. HFRS accounts for more than 20,000 cases annually in Asia and Europe, with an associated case fatality rate ranging from 0.1% to 15% [4]. In contrast, HPS is found exclusively in South America and North America, but it poses a significantly higher case fatality rate of up to 50% [5]. The majority of HPS patients succumb within 1 to 2 days after hospital admission [6]. Therefore, orthohantaviruses poses a significant global public health challenge [4].

Reservoir hosts of orthohantaviruses are widely distributed in nature, presenting a challenge for eradication efforts [4]. Orthohantaviruses have been known to be transmitted among various murid and cricetid rodent species in both the Eastern and Western hemispheres, with *Apodemus*, *Clethrionomys*, and *Rattus* serving as primary reservoir hosts [2,7,8]. As different kinds of orthohantaviruses may share one host, the virus–host species specificity paradigm could be blurred and thus contribute to the virus’s spread and likely to the evolution of closely related pathogens. Orthohantaviruses are typically transmitted to humans via urine, feces, saliva, and aerosols generated by reservoir hosts; however, transmission through animal bites is infrequent [9]. Human-to-human transmission of Andes virus (ANDV) has been documented [10,11,12,13], although this mode of transmission lacks widespread consensus [14].

The pathogenic mechanism underlying orthohantavirus infection remains incompletely understood, and there are currently no specific antiviral treatments. Therefore, the development of more effective vaccines is crucial for both prevention and control strategies against orthohantavirus infection. In recent decades, various vaccine approaches have been investigated, including attenuated vaccines, inactivated vaccines, virus-like particles, recombinant proteins, and gene vaccines. Currently, no orthohantavirus vaccine has received approval from the WHO [15]. However, scientists in China, South Korea, the United States, Russia, and other countries have conducted extensive research on vaccine development, efficacy, and safety. Notably, several vaccines developed in China and South Korea have been in use within these countries for many years. We present a comprehensive overview of the progress made in orthohantavirus vaccine research, as well as the benefits, drawbacks, and challenges associated with different types of vaccines.

### 1.1. The Growing Demand for Vaccines Against Orthohantaviruses

**Figure 1:**
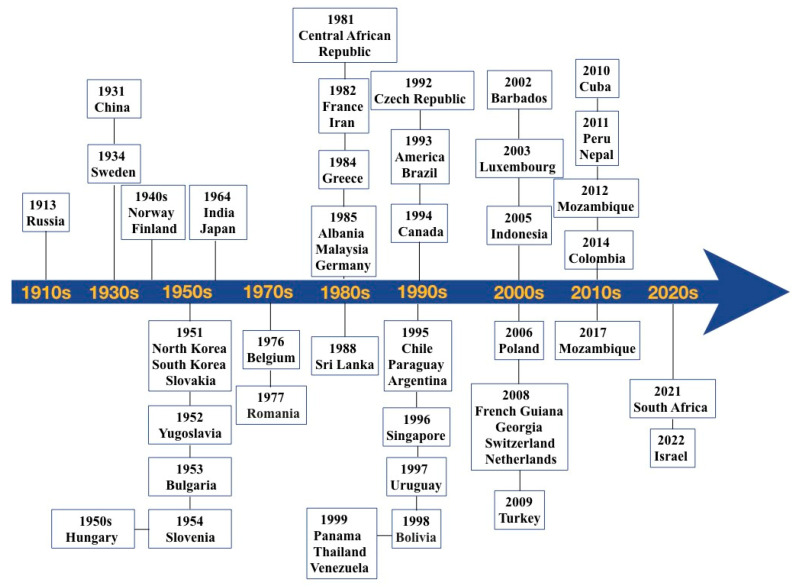
The worldwide distribution of orthohantavirus infection. Data published by PubMed and the International Travel Health Care Center (ITHCC). Sweden [22], China [23], North Korea/South Korea [24], Slovakia [25], Yugoslavia [26], Bulgaria [27], Slovenia [28], India [29], Norway [29], Finland [30], Romania [31], Japan [32], Belgium [33], Greece [34], Albania [35], Malaysia [36], Thailand [37], Central African Republic [38], France [39], Sri Lanka [40], Czech Republic [41], America [24], Brazil [42], Canada [43], Netherlands [44], Chile [45], Paraguay [46], Argentina [47], Singapore [48], Germany [49], Panama [50], Barbados [51], Indonesia [52], Luxembourg [53], Poland [54], French Guiana [55], Georgia [56], Switzerland [57], Turkey [58], Cuba [59], Iran [60], Colombia [61], Mozambique [62], Nepal [63], South Africa [64], Israel [65], Venezuela [55], Bolivia [55], Peru [55], Uruguay [47], Hungary [66].

Hemorrhagic fever conditions have been clinically presented since the *Yellow Emperor’s Internal Canon* in imperial China during the Warring States period (475–221 BCE) [16], and were then noted during World War 1 in Asian and European countries [2,17]. More scientifically accurate records of the disease have been available since approximately 1913 in Russia [2]. During the Korean War in 1951, hundreds of U.S. military personnel were diagnosed with “Korean hemorrhagic fever,” also called “epidemic hemorrhagic fever,” causing international concern [18]. Since then, numerous analogous cases have emerged globally, and the disease was subsequently assigned the standardized name HFRS by the World Health Organization (WHO) in 1982 [2]. Currently, nephropathia epidemica (NE), a mild form of HFRS, remains a prevalent zoonosis in Russia [19] and Finland [20]. The first cases of HPS were recognized in the U.S. in 1993 [21], and since that time, the syndrome has been recognized in a number of other Western Hemisphere countries (Figure 1).

Recent decades have seen increased likelihood of contact with orthohantavirus-infected wildlife, primarily rodents, due to human encroachment on their habitats coupled with climate change and landscape modifications [67,68]. The resurgence of global HFRS outbreaks in 2017 has refocused attention on this long-standing public health issue. Additionally, ongoing global conflicts may expose more individuals, including soldiers, to orthohantaviruses. As climate change and deforestation drive rodent population growth, the incidence of human orthohantavirus infections is expected to rise, transcending previous geographical limitations [69,70]. Consequently, preventing and managing orthohantavirus infections has become imperative. Projections indicate a growing global demand for orthohantavirus vaccines [71]. Given the heightened risk of outbreaks and the high mortality rates associated with both HFRS and HPS, there is an urgent need to strengthen medical preparedness for orthohantavirus infections.

### 1.2. The Immunological Basis for Rational Vaccine Design

**Figure 2:**
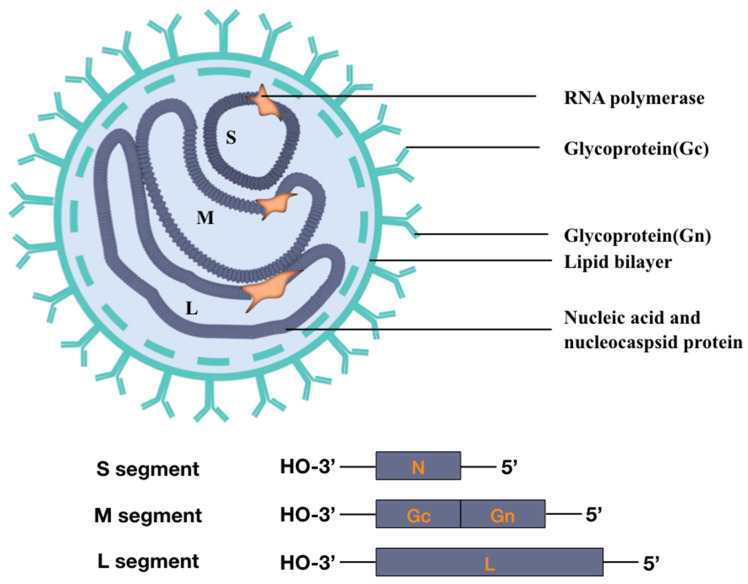
Schematic representation of the orthohantavirus virion. The orthohantavirus particle contains the viral RNA (vRNA) genome, which comprises small, medium, and large ORFs. These are encapsidated by the nucleocapsid protein. The outer part of the virion glycoprotein is composed of Gn and Gc. The viral genome is replicated and transcribed by RNA polymerase. The diameter of the virus is approximately 80–120 nm.

Orthohantavirus is an enveloped virus with a tri-segment negative-sense RNA genome [72]. The virus particle is oval or spherical, measuring 80–120 nm in diameter. The RNA segments of the orthohantavirus genome are designated as small (S, 1.8–2.1 kb), medium (M, 3.7–3.8 kb), and large (L, 6.5–6.6 kb). The conserved 3’- and 5’-ends of the segments may form a panhandle structure to act as the viral promoter [73]. The S and M segments encode the nucleocapsid protein (NP) and the envelope glycoprotein (GP, a polyprotein of 1133–1158 aa residues composed of Gn and Gc), respectively. The L segment encodes the RNA-dependent RNA polymerase (RdRp) [74]. Each virus particle usually comprises equivalent quantities of genomic RNA, featuring a solitary viral RdRp molecule bound to each RNA segment. These RNA segments are encapsulated by NP proteins, thereby constituting ribonucleoprotein complexes. The RdRp is responsible for both the transcription and replication processes of the virus [75]. RNA polymerase cleaves cellular mRNA, forming capped primers, which are protected from degradation by bounding viral NP. NP consists of approximately 433 amino acid residues (about 50 kDa in size) with high conservatism. GP may interact with host cellular surface proteins, facilitating cellular entry [75]. As mentioned earlier, orthohantavirus has the capacity for reassortment. Notably, the resultant reassorted viruses consistently preserved the S and L segments from one parental orthohantavirus, whereas the M segment was acquired from the alternative parent [76] (Figure 2).

GPs play critical roles in initiating humoral immunity and can stimulate the production of virus-specific neutralizing antibodies [77,78,79]. Monoclonal antibodies targeting GPs have demonstrated virus-neutralizing activity, both in vitro and in vivo [80,81]. However, GPs have weak immunogenicity, characterized by a delayed antibody response and low antibody titers [82]. Among GPs, the Gc glycoprotein is responsible for directing viral fusion activity and is classified as a class II viral fusion protein [83]. Antibodies against Gc can prevent viral entry into the host.

In contrast to GPs, NP has the strongest immunogenicity and can activate antibody-dependent cytotoxic T cells [84,85]. NP is more conserved and induces a highly cross-reactive antibody response [86]. It has been reported that immunization with NP can induce a protective immune response that increases the survival of mice following challenges with a lethal dose of orthohantavirus [87]. Therefore, NP has become an attractive candidate antigen for orthohantavirus vaccine. Presently, there is no evidence that the protein encoded by the L segment induces an immune response during orthohantavirus infection.

**Figure 3:**
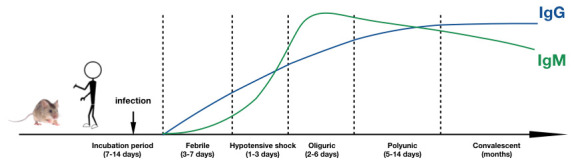
Temporal dynamics of orthohantavirus-specific immunoglobulins during the onset of HFRS. This figure references previously published Chinese literature and one review [91].

Orthohantavirus infection is marked by high levels of virus-specific IgM detected at disease onset in humans. After initial infection, the viral load increases in the host, stimulating the production and rising titers of virus-specific antibodies. Serum virus-specific IgM usually peaks 7–11 days after onset and declines during the convalescent phase, usually coinciding with the rise of IgG [88]. Orthohantavirus can induce early IgG1 responses that increase with disease progression. IgG2 usually remains unchanged during infection [89]. IgG3 significantly increases in the clinical course, peaking during the convalescent phase and then declining gradually decades after infection (Figure 3). Orthohantavirus-specific antibodies, especially IgG, can persist for up to 34 years after natural infection [90]. No correlation has been found between IgG subclass levels and disease severity.

In orthohantavirus infection, cellular immunity may be part of the complex immune response in affected human organs. Both HFRS and HPS are marked by robust cytotoxic T lymphocyte (CTL) responses [92]. During orthohantavirus clearance, lasting CD8+ T-cell responses occur [93,94] without increases in neutralizing antibodies titers, which are mediated by cytotoxic CD8+ T cells. Orthohantavirus-infected cells are protected from cytotoxic lymphocyte-mediated apoptosis by high levels of granzyme B and perforin [15,95]. The immune response to hantaviruses is characterized by a balanced Th1/Th2 response, as evidenced by cytokine profiles in sera. This type of response is aimed at promoting a less harmful and more controlled immune response [96]. In HPS, inactivation of Tregs not only reduced viral RNA in lungs and virus shedding in rats but also decreased lung lesions [97]. Orthohantaviruses can be effectively combated through the activation of cytotoxic T cells and the subsequent generation of cross-protection. Therefore, in evaluating vaccine effects, in addition to neutralizing antibodies, the following cellular immune indicators are included: the proliferation ability of specific T lymphocytes, the levels of cytokines secreted by specific T lymphocytes, the killing ability of CD8+ CTLs on orthohantavirus-infected cells, and particularly the number and function of memory T cells, which are for long-term immune protection.

## 2. Vaccines Against Orthohantaviruses

### 2.1. Inactivated Vaccines

An inactivated vaccine was the pioneering orthohantavirus vaccine used to prevent HFRS in endemic regions. Inactivated vaccines are generated by subjecting a pathogen to heat or chemical treatments, which prevent replication while preserving immunogenicity, allowing the human immune system to recognize and respond to the pathogen. This methodology is well established, efficient, and cost-effective, leading to widespread adoption. Since the discovery of HTNV in 1976, researchers have explored the potential of orthohantavirus vaccines, primarily focusing on inactivated vaccines. However, vaccine development was limited due to challenges in virus isolation, large-scale viral culture, and biosecurity measures.

In 1988, South Korea successfully developed an inactivated vaccine named Hantavax using cultured brain cells of suckling mice infected with HTNV [98]. High-risk individuals, such as military personnel and rural residents who frequently encounter rodents, receive this inactivated vaccine [99]. The South Korean military implemented additional preventive measures, including avoiding training sites in areas with suspected elevated HFRS risks, rodent eradication, and clearing bushes or tall grass in nearby environments [100]. Following these interventions, the incidence of HFRS within the military exhibited a declining trend, starting from the 2000s [101]. A trial enrolled 3900 subjects to evaluate Hantavax efficacy, observing no confirmed HFRS cases among 1900 Hantavax vaccinees, while 20 cases were observed among 2000 non-vaccinated controls [102]. However, a small-scale controlled study indicated that Hantavax elicited only low levels of neutralizing antibodies and provided limited protection [103]. A retrospective study suggested that while Hantavax might reduce the severity of acute kidney injury, it did not significantly prevent the progression of HFRS [101]. Comparative analysis revealed distinct patterns of disease severity between a Hantavax-vaccinated group and an unvaccinated group [104]. To enhance the efficacy of vaccination, in 2018, the Korean Ministry of Food and Drug Safety officially announced an increase in the total dosage regimen of the inactivated vaccine from three to four doses [105]. A multicenter phase III clinical trial of a modified schedule (3 + 1 vaccination at 0, 1, 2, and 13 months) (NCT 02553837) revealed that Hantavax demonstrated a high seroconversion rate following the three-dose initial vaccination series. Furthermore, an additional dose administered 11 months later resulted in significant booster effects [106]. Despite ongoing debates about the protective efficacy and cost-effectiveness of vaccines since their widespread implementation [103], the use of inactivated vaccines for prevention in high-incidence areas of HFRS remains a crucial and viable option for public health [107].

In 1993, China approved its first inactivated orthohantavirus vaccine for clinical use, and in 1995, the vaccination program was launched in regions with a high incidence of HFRS [99,108]. A bivalent inactivated vaccine targeting SEOV and HTNV was developed in 1994 and subsequently incorporated in the Chinese Expanded Program on Immunization (EPI) in 2003. This bivalent vaccine is administered annually to residents aged 16–60 years in endemic areas, with approximately 2 million doses distributed nationwide [107,108]. Following the introduction of this vaccine, the incidence of HFRS decreased from 37,814 cases in 2000 to 11,248 cases in 2007 [108]. To date, four vaccines have been produced in China: three monovalent Hantaan vaccines (gerbil kidney cell vaccine, hamster kidney cell vaccine, and mouse brain vaccine) and one bivalent gerbil kidney cell vaccine against HTNV and SEOV [109]. The inactivated HTNV vaccine has demonstrated the potential to induce high titers of neutralizing antibodies and stimulate specific humoral and cellular immune responses in BALB/c mice against the prevalent HTNV strain [110]. A recent model suggests that enhancing vaccination coverage and reducing rodent populations, particularly wild mice, are critical strategies for decreasing HFRS infections [111]. The sustained implementation of bivalent inactivated vaccine programs in high-prevalence regions in China has been a critical strategy to reduce the incidence of HFRS. Over the last five years, this approach has contributed to maintaining the annual number of HFRS cases below 10,000.

Russia reports approximately 7300 cases of HFRS annually, with an overall case fatality rate (CFR) of 0.4%. PUUV is the causative agent for nearly all HFRS cases identified in western regions of Russia [112]. In 2020, Russian researchers reported the results of preclinical studies for a candidate polyvalent HFRS vaccine. The inactivated polyvalent vaccine, based on Puumala (strain PUU-TKD/VERO), Hantaan (strain HTN-P88/VERO), and Sochi (strain DOB-SOCHI/VERO) viruses, remains in the preclinical phase [113]. A recent study demonstrated that different adjuvants exert distinct effects on the immunogenicity of the PUUV vaccine [114]. Compared to the traditionally used aluminum hydroxide, both the B subunit of heat-labile enterotoxins and plant virus-based spherical particles significantly enhance the humoral immune response elicited by the vaccine. Low-endotoxicity lipopolysaccharide not only boosts the immune response but also maintains the vaccine’s immunogenicity for at least one year of storage [114]. These findings suggest potential improvements for inactivated vaccines; however, no new inactivated vaccines are expected to become clinically available in the near future.

### 2.2. Viral Vector-Based Vaccines

Viral vector-based vaccines, particularly those expressing orthohantavirus antigens, have demonstrated the ability to elicit both humoral and cellular immune responses without the need for adjuvants. Preclinical animal models have shown that this approach can result in a robust and favorable immune response against orthohantavirus infection. Recombinant adenoviruses (AdVs) expressing GnS0.7, a fusion protein containing Gc and truncated NP, have been constructed and shown to induce strong HTNV-specific immune responses in mice [115,116]. Moreover, given that heat-shock proteins (HSPs) exhibit adjuvant properties when administered as fusion proteins to boost vaccination efficacy, a recombinant AdV vector named rAdV-GnS0.7- pCAG-HSP70C was constructed. This vector was engineered by fusing the C-terminal region of the HSP70 gene with the HTNV Gn protein and a 0.7-kilobase fragment of the NP gene [117]. Animal protection experiments demonstrated that this recombinant adenovirus enhanced humoral and cellular immune response [117]. Non-replicating AdV vectors expressing Andes virus (ANDV) NP or GP have been constructed to protect Syrian hamsters from lethal ANDV infection by producing strong cytotoxic T-lymphocyte responses directed to orthohantavirus proteins and without detectable neutralizing antibodies [118]. Syrian hamsters immunized with a single dose of recombinant vesicular stomatitis virus (VSV) vector-based vaccine expressing ANDV surface glycoprotein were protected against lethal ANDV infection challenges [119]. A replication-competent recombinant VSV vaccine expressing the GP of ANDV instead of the native VSV GP has also been developed [120]. This vaccine was highly efficacious in the Syrian hamster model of HPS within 6 months, but the protective effect decreased one year later [120]. Recombinant vaccines constructed respectively against ANDV and SNV viruses were found to produce cross-immunity against each other [121]. In 2024, a recombinant (r) rVSV-HTNV-GP was also engineered and provided longer-term protection against HTNV infection than inactivated vaccines [122].

The emergence of viral vector-based vaccines, such as recombinant adenovirus type 5 (Ad5)-vectored COVID-19 vaccine [123], has sparked concerns about the influence of pre-existing immunity to the vaccine vector on vaccine efficacy [124]. This issue became especially pertinent in the aftermath of the COVID-19 pandemic and overlapping endemic areas. A previous study indicated that pre-existing vaccinia immunity can diminish the efficacy of recombinant vaccinia vaccines for HTNV [125]. Similarly, pre-existing immunity to the AdV vector has been observed to affect both immunogenicity and efficacy, particularly at lower vaccine doses [126]. Repeated immunization can improve vaccine protective efficacy, but whether consecutive immunization of a vaccine based on the same viral vector would be effective in the same population due to pre-existing immunity is uncertain. Investigating the pre-existing immune status of the viral vector in the population in advance may help select more advantageous virus vectors, such as AdV 35 vs. AdV 5, and using higher vaccine doses may overcome pre-existing immunity to the virus vector [126]. Heterologous sequential immunization can utilize different antigen types, delivery platforms, and administration routes to induce multifaceted immune responses, which may be a practical strategy [127].

There is a need to address safety issues associated with the administration of replicating vaccines in humans [128]. One concern is the potential neurovirulence of VSV. The possibility of generating a virulent phenotype through recombination of the genomes of these virus vectors and circulating viruses cannot be ignored [129]. Another consideration is vaccine safety in immunocompromised individuals, as they can replicate efficiently. In regard to large-scale use of viral vector-based vaccines, severe and unexplained conditions must be considered. For instance, a phase III clinical trial of a COVID-19 vaccine was paused due to AdV-induced immune thrombosis and thrombocytopenia syndrome [130].

### 2.3. Virus-like Particles

Virus-like particles (VLPs) are composed of repetitive viral structural proteins with inherent self-assembly characteristics. They resemble natural virus particles, but lack infectious genetic material [131]. VLPs can elicit potent antiviral humoral and cellular immune responses because they can mimic the virus and are efficiently taken up, rapidly internalized, and processed by antigen-presenting cells (APCs) [132,133,134]. VLPs have been developed for several viruses, such as hepatitis B [132], HIV-1 [133], and HPV [134]. Therefore, VLPs have garnered great attention in the development of hantavirus vaccines.

Studies have demonstrated that chimeric VLPs devoid of orthohantavirus nucleic acids can elicit an orthohantavirus-specific immune response from the host. VLPs were engineered using hepatitis B virus (HBV) capsids, incorporating a 120-amino acid segment from the NP of highly pathogenic DOBV and HTNV. Immunization with these chimeric VLPs induced a high-titer, cross-reactive NP-specific antibody response in both BALB/c and C57BL/6 mice [135,136]. Additionally, a 99-amino acid-long segment of Gc protein from PUUV was inserted into the major capsid protein VP1 of hamster polyomavirus to create VLPs. These chimeric VLPs induced an efficient insert-specific antibody response in immunized mice [137]. Recombinant HTNV-VLPs were produced by co-expressing HTNV-NP and GP in Chinese hamster ovary (CHO) cells using a human IgG heavy chain expression vector. These VLPs demonstrated that intramuscular and subcutaneous immunizations could effectively stimulate specific humoral and cellular responses against HTNV-NP and GP comparable to those induced by an inactivated bivalent orthohantavirus vaccine [132,138]. Furthermore, reconstructed VLPs incorporating CD40 ligand or granulocyte–macrophage colony-stimulating factor (GM-CSF) were created to enhance immunogenicity. The study showed that these modified VLPs significantly increased neutralizing antibody titers against HTNV and provided stable, long-term protection for up to 6 months post-immunization in mice [139].

VLPs have demonstrated potential for controlling orthohantavirus infection [140]. VLPs lack genetic material, which reduces the risk of adverse effects and is intrinsically safer than attenuated virus vaccines [140]. However, VLPs may degrade more rapidly than those derived from live organisms. Unlike attenuated organisms, VLPs lack the inherent pro-inflammatory properties of live vaccines. Therefore, optimizing VLP stability and effectiveness remains a subject for further study. Combining VLPs with immunopotentiators may be a promising approach to enhance vaccine efficacy.

### 2.4. Subunit Vaccines

Subunit vaccines contain specific microbial components, such as proteins or surface antigens, that can induce neutralizing antibodies. Recombinant protein subunit vaccines avoid potential issues with purified macromolecule subunit vaccines, including co-purification of undesired contaminants or reversal of toxoids to toxigenic forms and interference between multivalent formulation components [141,142,143]. Thus, recombinant DNA technology has been proposed for expressing orthohantavirus proteins for hantavirus subunit vaccine development [144,145]. Recombinant DOBV nucleocapsid proteins (rNPs) in mice induced a strong NP-specific IgG response and mixed Th1/Th2-cell involvement [146]. DOBV rNPs conjugated with recombinant rP40 (the Klebsiella pneumoniae outer membrane protein A) showed higher NP-specific antibody responses. Immunogenicity of PUUV rNP–rP40 was characterized [147]. Recombinant PUUV proteins were expressed in E. coli 200. The truncated construct, P40-Puu118, gave high antibody titers after two doses and 100% protection after three doses, suggesting potential as a subunit vaccine against PUUV [148].

Recent years have witnessed significant advancements in both immune protection technology and bioinformatics, particularly high-throughput sequencing technology, accelerating the development of multi-epitope subunit vaccines through the reverse-vaccinology approach. In 2022, Ismail screened 340 epitopes from the Virus Pathogen Database and Analysis Resource (ViPR), identifying 10 epitopes for potential inclusion in a potent multi-antigenic epitope subunit vaccine. These epitopes were linked through specific GPGPG linkers to create a computationally designed vaccine [149]. This vaccine was then computationally combined with three different adjuvants to stimulate immune responses. A major limitation of that study is the lack of experimental validation.

### 2.5. Nucleic Acid Vaccines

The third-generation nucleic acid vaccine has shown promise in inducing both humoral and cellular immunity without requiring additional immunization, making it a potentially effective vaccination strategy for a variety of viral infections [150]. This approach has demonstrated good safety, as the vaccines are replication-defective and cannot revert to virulence or transmit from person to person or into the environment [151]. Moreover, the nucleic acid vaccine offers an easy method for constructing multivalent vaccines [91].

A DNA vaccine expressing HTNV M gene products, G_1_ and G_2_, was developed [152,153]. It was shown that this vaccine induced high levels of neutralizing antibodies and provided complete protection against HTNV, SEOV, and DOBV infections in hamsters [154]. The phase I clinical trial demonstrated the safety of DNA vaccines encoding the HTNV or PUUV M-segment delivered by electroporation separately or as a mixture [154]. The mixture showed safety and induced high-frequency immune responses in the phase IIa clinical trial [155]. A DNA vaccine based on the ANDV M gene and a DNA plasmid encoding both the ANDV and HTNV M genes induced high-titer neutralizing antibodies in rabbits and nonhuman primates [156,157]. Recently, a DNA vaccine developed using the complete M gene of SNV was engineered, and it successfully elicited high-titer neutralizing antibodies [71]. These studies suggest that DNA vaccines can provide cross-protection against various species of orthohantaviruses [152,158]. Interestingly, the HPS vaccine elicited immune responses against SNV and ANDV, whereas it did not induce reactions against HTNV and PUUV. In contrast, the HFRS vaccine triggered responses to HTNV and PUUV, but was ineffective against ANDV and SNV. These findings highlight distinct differences in reactivity among various orthohantavirus species [71].

A DNA vaccine was constructed incorporating the fusion of HTNV NP and GP with the extracellular region of cytotoxic T lymphocyte-associated antigen 4 (eCTLA-4), aimed at targeting antigen-presenting cells (APCs). This eCTLA-4 fusion approach led to improved antibody and cellular immune responses in mice [159]. Moreover, a DNA vaccine was developed that targets the hantavirus glycoprotein N-terminal (Gn) or Gc and is presented to the major histocompatibility complex class II compartment by fusing the glycoproteins with lysosome-associated membrane protein 1 (LAMP1) [160,161]. By altering the antigen-processing pathway for major histocompatibility complex II, LAMP1 contributed to enhanced long-term humoral immunity [162].

Nucleic acid vaccines, particularly DNA vaccines, are at the forefront against orthohantaviruses, with several in clinical trials (Table 1). RNA vaccines have also shown promise. Studies have compared non-modified and modified mRNA vaccines against ANDV, evaluating immunogenicity, germinal center induction, antibody responses, and protective efficacy. The results showed that both versions provided protection without significant differences in immunogenicity or protection in rodents [163]. Three novel nucleic acid vaccine candidates—mRNA, naked DNA, and DNA-LNP encoding HTNV glycoproteins—were assessed in mice. All candidates induced robust and sustained immune responses, matching the protective efficacy of inactivated vaccines. Notably, mRNA triggered stronger T-helper 1 cell responses, while DNA-LNP produced higher neutralizing antibodies compared to inactivated vaccines [164].

## 3. Prospect of Vaccine Development and Application

Historically, inactivated vaccines have been the dominant approach for designing orthohantavirus vaccines, due to their relative ease of establishment. However, concerns about nondurable immunity and complex immune components have prompted exploration of alternative vaccine types, including viral vector-based vaccines, virus-like particles, and subunit vaccines. Advances in bioinformatics and computational biology have significantly accelerated this development [165,166,167]. Comprehensive computational analyses of orthohantavirus motifs and codon usage have provided insights into host immune regulation and evolutionary history, aiding identification of conserved epitopes and antigenic regions crucial for robust immune responses [168,169]. Molecular docking and dynamic simulations evaluate binding affinity between vaccine candidate proteins and host immune receptors [170], while immune simulation analysis can predict natural immune responses [165,171]. Several multi-epitope subunit vaccines for cross-protection against orthohantaviruses, such as HTNV and PUUV, have been developed based on immunoinformatic approaches [149,165,166,170,171,172,173]. However, all computationally designed vaccines require experimental validation before clinical application.

In addition to vaccine types and selected epitopes, different adjuvants and immunization strategies are crucial. Compared to traditional mineral adjuvants, particulate adjuvants, and protein adjuvants, lipopolysaccharide has demonstrated more pronounced stimulation of immunogenicity [114]. An immunoinformatic-designed vaccine tested three different adjuvants—TLR4-agonist adjuvant, β-defensin, and 50S ribosomal protein L7/L12—aiming to enhance immune responses and evaluate the vaccine’s efficacy with each adjuvant [149]. Various vaccination routes, including intramuscular injection and mucosal vaccination, have been explored [174]. Moreover, an effective immunization schedule is essential, especially when a single dose of vaccine does not provide adequate protection. For instance, in March 2018, the Korean Central Pharmaceutical Affairs Council of the Ministry of Food and Drug Safety (MFDS) increased the number of Hantavax inoculations from three to four. Heterologous sequential immunization may serve as a practical strategy to boost immune efficacy.

Testing and approving orthohantavirus vaccines presents unique challenges due to the limited patient population and difficulties in conducting large-scale clinical trials. For other rare diseases, researchers often rely on adaptive trial designs to assess vaccine efficacy, allowing modifications based on interim results or surrogate endpoints like immune response markers. However, these markers are not universally recognized as reliable surrogates for clinical endpoints. Initially, the seropositivity rates from four major methods used to measure vaccine immunogenicity varied significantly [106]. Moreover, maintaining immunogenicity does not necessarily equate to vaccine effectiveness, which remains to be validated. Identifying a reliable surrogate endpoint could enable regulatory agencies such as the FDA and EMA to employ accelerated approval pathways, contingent on post-marketing surveillance, thereby expediting access to these vaccines.

## 4. Conclusions

As a significant global public health concern, the research and development of orthohantavirus vaccines hold considerable practical importance. While notable advancements have been made across various vaccine platforms, including inactivated, subunit, viral vector, VLP, and nucleic acid vaccines, several challenges remain. These include inadequate immunogenicity, limited broad-spectrum protective efficacy, and complexities in manufacturing and distribution. Moving forward, by refining vaccine design, enhancing vaccination strategies, establishing rigorous evaluation criteria, and fostering international collaboration, it is anticipated that orthohantavirus vaccine research will achieve critical breakthroughs. Ultimately, the development of a safe and effective vaccine will provide a robust tool for preventing and controlling orthohantavirus infections, thereby making a substantial contribution to global public health security.

## Figures and Tables

**Table 1:** Existing DNA vaccines in clinical trials.

NCT Number	Title	Intervention	Type	Funder	Age	Number Enrolled	Date	Status	Major Outcomes
02776761	A Single-blind Study to Evaluate the Safety, Tolerability, and Immunogenicity of a Hantaan Puumala Virus DNA Vaccine	Hantaan vaccine, Puumala vaccine, mixed Hantaan/Puumala vaccines	Phase I: single-center, randomized, single-blinded	U.S. Army Medical Research and Development Command	18–49	27	30 August 2016	Complete	Neutralizing antibody responses: 7/7 (100%) of HTNV vaccines; 6/6 (100%) of PUUV vaccines; 4/9 (44%) seroconversion against both viruses of mixed vaccines
02116205	Phase 2a Immunogenicity Study of Hantaan/Puumala Virus DNA Vaccine for Prevention of Hemorrhagic Fever	Hantaan/Puumala vaccines	Phase IIa: randomized, double-blind, dose-optimizing	U.S. Army Medical Research and Development Command	18–49	130	9 July 2014	Complete	
04333459	Safety and Immunogenicity of a Hantaan Virus DNA Vaccine and a Puumala Virus DNA Vaccine, For the Prevention of Hemorrhagic Fever With Renal Syndrome	Hantaan Vaccine/Puumala Vaccine	Phase II: randomized, double-blind	U.S. Army Medical Research and Development Command	18–49	132	23 August 2021	Recruiting	
03682107	Andes Virus DNA Vaccine for the Prevention of Hantavirus Pulmonary Syndrome Using the PharmaJet Stratis(R) Needle-Free Injection Delivery Device	Andes virus vaccine	Phase I: randomized, placebo controlled, double-blind, dose escalation	National Institute of Allergy and Infectious Diseases	18–49	48	19 February 2019	Complete	98% and 65% of subjects had at least 1 local or systemic solicited adverse event
03718130	Combination HTNV and PUUV DNA Vaccine	Vaccine–device combination	Phase I: randomized	U.S. Army Medical Research and Development Command	18–49	72	11 October 2019	Active, not recruiting	
01502345	Study to Evaluate the Safety, Tolerability, and Immunogenicity of Hantaan and Puumala Virus DNA Vaccines	Vaccine–device combination	Phase I: randomized	U.S. Army Medical Research and Development Command	18–49	31	January 2012	Complete	

## Abbreviations

The following abbreviations are used in this manuscript:

Ad5	adenovirus type 5
APCs	antigen-presenting cells
ANDV	Andes virus (*Orthohantavirus andesense*)
CHO	Chinese hamster ovary
COVID-19	coronavirus disease 2019
CTL	cytotoxic T lymphocyte
DOBV	Dobrava-Belgrade virus (*Orthohantavirus dobravaense*)
EPI	Expanded Program on Immunization
GM-CSF	granulocyte–macrophage colony-stimulating factor
GP	glycoprotein
HFRS	hemorrhagic fever with renal syndrome
HPS	hantavirus pulmonary syndrome
HSPs	heat-shock proteins
HTNV	Hantaan virus (*Orthohantavirus hantanense*)
NE	nephropathia epidemica
NP	nucleocapsid protein
PUUV	Puumala virus (*Orthohantavirus puumalaense*)
RdRp	RNA-dependent RNA polymerase
SEOV	Seoul virus (*Orthohantavirus seoulense*)
SNV	Sin Nombre virus (*Orthohantavirus sinnombreense*)
VLPs	virus-like particles
VSV	vesicular stomatitis virus
WHO	World Health Organization
